# Anti-Biofilm Enzymes-Assisted Antibiotic Therapy against Burn Wound Infection by Pseudomonas aeruginosa

**DOI:** 10.1128/aac.00307-23

**Published:** 2023-06-05

**Authors:** Yixin Zhang, Xiaolong Liu, Huamei Wen, Zhongle Cheng, Yanyu Zhang, Haichuan Zhang, Zhongwen Mi, Xinjiong Fan

**Affiliations:** a School of Basic Medical Sciences, Anhui Medical University, Hefei, Anhui, China; b University of Science and Technology of China, Hefei, Anhui, China; c College & Hospital of Stomatology, Anhui Medical University, Key Lab. of Oral Diseases Research of Anhui Province, Hefei, China; d The First Affiliated Hospital of Anhui Medical University, Hefei, Anhui, China

**Keywords:** anti-biofilm enzyme, *Pseudomonas aeruginosa*, burn wound infection, tobramycin, combination therapy

## Abstract

Pseudomonas aeruginosa can form biofilms at the site of burn wound, leading to infection and the failure of treatment regimens. The previous *in vitro* study demonstrated that a combination of the quorum-quenching enzyme AidH_A147G_ and the extracellular matrix hydrolase PslG was effective in inhibiting biofilm and promoting antibiotic synergy. The aim of the present study was to evaluate the efficacy of this combination of enzymes in conjunction with tobramycin in treating burn wound infected with P. aeruginosa. The results showed that this treatment was effective in quorum-quenching and biofilm inhibition on infected wounds. Compared with the tobramycin treatment only, simultaneous treatment with the enzymes and antibiotics significantly reduced the severity of tissue damage, decreased the bacterial load, and reduced the expression of the inflammatory indicators myeloperoxidase (MPO) and malondialdehyde (MDA). Topical application of the enzymes also reduced the bacterial load and inflammation to some extent. These results indicate that the combined-enzyme approach is a potentially effective treatment for P. aeruginosa biofilm infections of burn wounds.

## INTRODUCTION

Burns are a major public health problem, and their treatment places an enormous burden on health care systems worldwide (1). One of the greatest challenges in burn care is bacterial infection, which delays healing and increases mortality (2, 3). Biofilm bacteria, being characterized by resistance to physicochemical aggression, immune responses, and antibiotics, are directly responsible for many of the therapeutic difficulties encountered in treating burns (4, 5). The opportunistic pathogen, Pseudomonas aeruginosa, has been identified as a particularly common cause of mortality and morbidity among burn patients (6, 7). Indeed, previous research has associated as much as 77% of burn wound mortality with P. aeruginosa (8). Most efforts to combat this organism have been directed at boosting the antimicrobial activity of a broad range of topically applied therapeutic agents, thereby facilitating the disinfection of wound sites (9, 10). Consequently, few new therapeutic options for burn patients have become available that treat biofilms specially.

Considerable effort has been made, however, to identify strategies for overcoming biofilm recalcitrance based on researchers’ understanding of the mechanisms of bacterial adhesion and biofilm formation in P. aeruginosa. Quorum-quenching, for example, has been shown to inhibit biofilm formation by altering the progression from the initial attachment of bacteria to the development of microcolonies and mature biofilm (11, 12). Moreover, quorum-quenching can downregulate the expression of the virulence factors associated with pathogenicity (13, 14). Acyl-homoserine lactones (AHLs) play a critical role in inhibiting the autoinducers of the quorum sensing systems Las and Rhl in P. aeruginosa (15, 16). In addition, inducing dispersal can expose biofilm bacteria to antibiotics and the host immune system (17). Enzymes that hydrolyze extracellular matrix components destabilize biofilm, making this approach an effective therapeutic strategy (18, 19).

Our previous *in vitro* experiments demonstrated that a combination of the acyl-homoserine lactone AidH_A147G_ and the glycosyl hydrolase PslG delivered superior results in inhibiting biofilm and increasing antibiotic sensitivity (20). Thus, the combined-enzyme therapy is an attractive therapeutic strategy for P. aeruginosa infection. Motivated by these preliminary observations, we sought to further explore the combined-enzyme therapy potential under *in vivo* conditions. Moreover, considering the seriousness and harmfulness of biofilm-associated infections to patients with burns, we established rat burn and surface infection model as a primary application of the enzyme therapy. The goals of this study were 4-fold: (i) to assess the biocompatibility of the combined enzymes by cell feasibility tests; (ii) to determine the direct effects of the combined enzymes by characterizing the quorum sensing system gene expression and biofilm formation; (iii) to assess the therapeutic effects of using the combined enzymes alone or in conjunction with antibiotics on wound healing; and (iv) to evaluate the results of the combined enzymes therapy on the inflammatory response. Our results demonstrated the potential of the combined enzymes to assist the treatment of burn infections. Because biofilm formation on burn wounds is a contributing factor to the failure of burn treatment regimens, it is critical that we find effective treatments for such infections.

## RESULTS

### Cytotoxicity assay.

**FIG 1:**
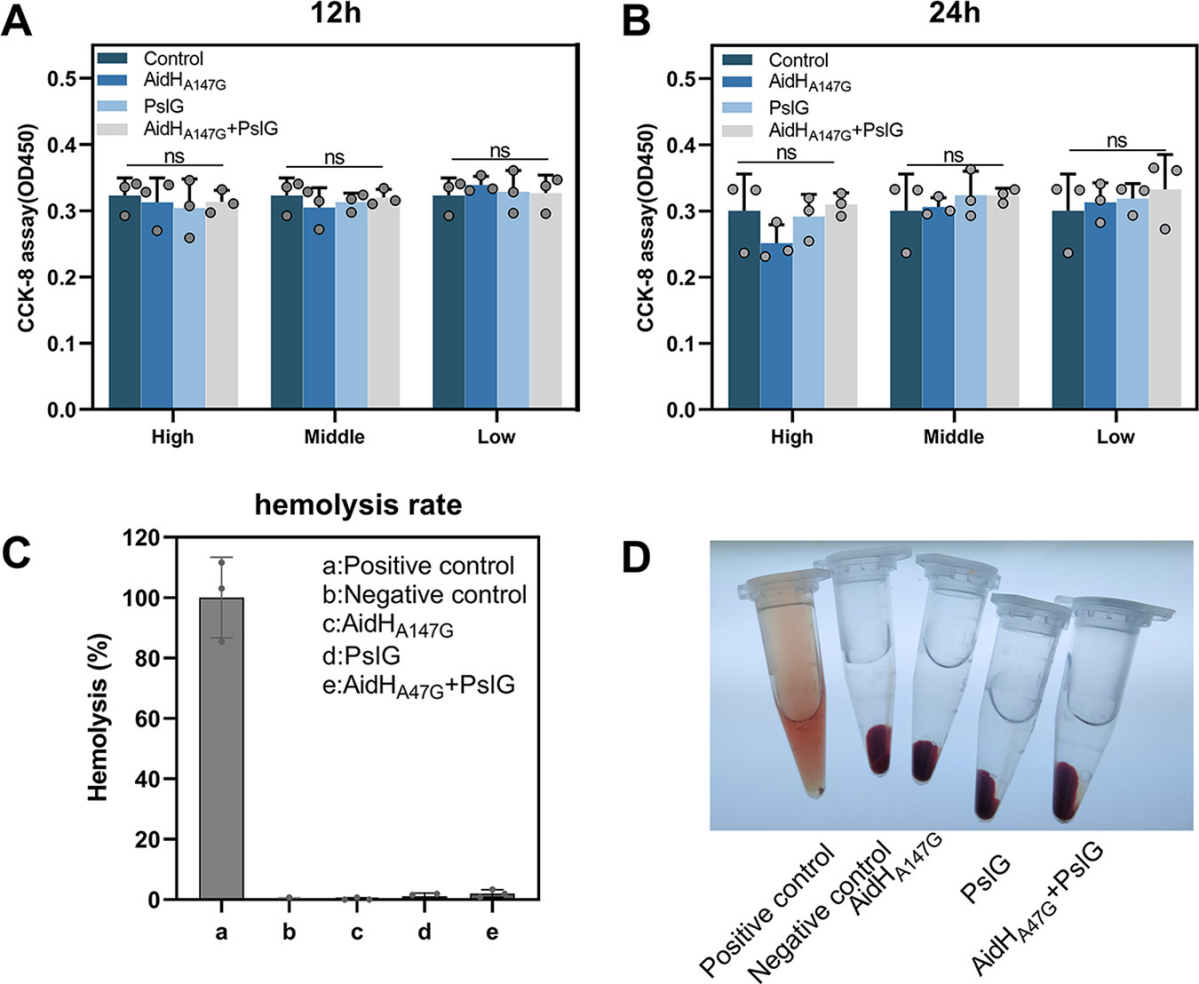
The result of cytotoxicity assay. (A) Viability of human dental pulp cells after 12 h treatment. (B) Viability of human dental pulp cells after 24 h treatment. (C) Hemolysis rate in different intervention groups. (D) Photo image showing the results of hemolysis rates for different treatments. NS, no significance.

We used human dental pulp cells to test whether the enzyme treatments had any toxic effects on mammal cells. The CCK8 assay revealed that treated with AidH_A147G_ and PslG, either alone or in combination, had minimal effect on the viability of human dental pulp cells (Fig. 1A and B). To evaluate cytotoxicity further, we determined whether the enzymes had any hemolytic effect on erythrocytes. Either macroscopically or spectrophotometrically, no obvious loss of intact erythrocytes was observed, suggesting that neither AidH_A147G_ nor PslG has no toxic effects on erythrocytes at the concentration of protein used (Fig. 1C and D). This result demonstrated that the combination of AidH_A147G_ and PslG had good potential as an agent for combating P. aeruginosa infection agent.

### Morphological analysis of burn wounds.

**FIG 2:**
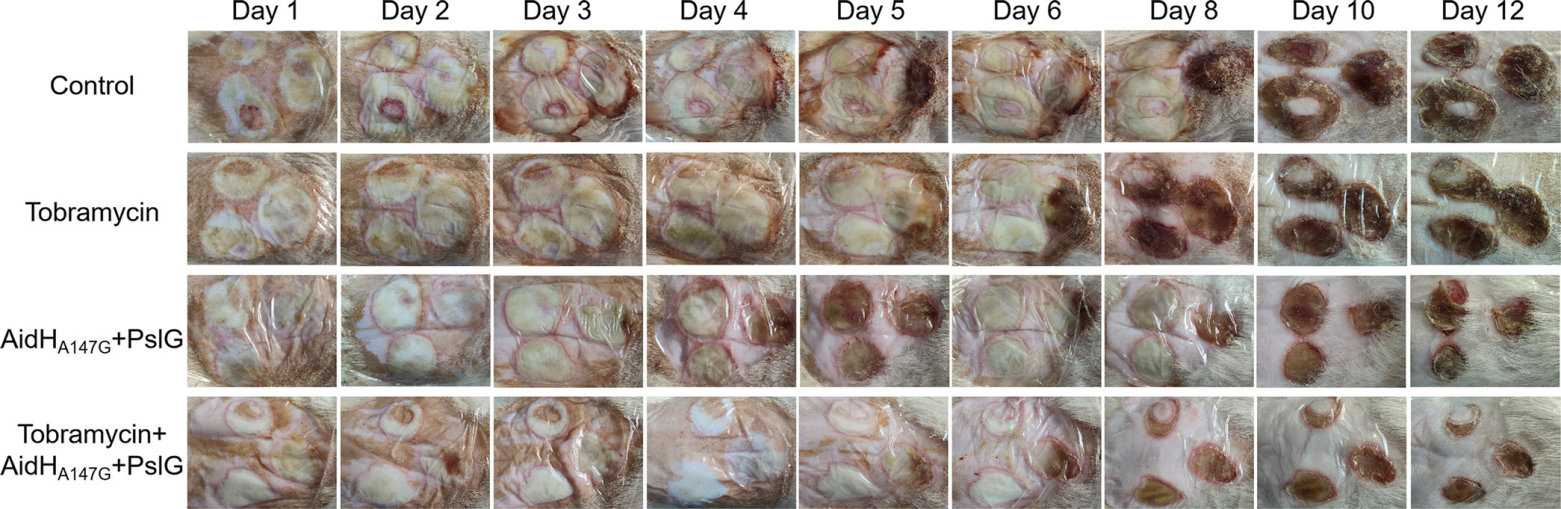
Representative visual appearance of the burn wound for different treatment groups on different days. The combined treatment group showed better wound recovery and thinner eschars compared to the other three groups.

On the first day after scalding, the appearance of the wounds showed the burned area to be clearly delineated from the adjacent skin and the wound to be swollen (Fig. 2). The discoloration of the wounds by the P. aeruginosa infection was evident in both the control and enzyme treatment groups. On the third day after scalding, hemorrhagic exudative signs on the surrounding tissues were observed in the control group. On the fourth to fifth days after scalding, the rats in all four groups started to develop scabs, but the nonscabbed part of the skin of those in the control group was still severely infected. On the sixth day after scalding, the wounds in the tobramycin and the combination treatment group (enzymes+tobramycin) were almost completely covered by scabs. Some rats in the enzyme treatment group showed recurrent wound infections (i.e., the infection becoming worse than on the previous day). After 10 days, the wounds were completely covered by scabs in all four groups. The combination treatment group wounds showed thinner eschars than those in the other three groups. Also after 10 days, we observed no significant variation in the rates of wound healing among the four groups. Overall, the combination treatment group showed the best therapeutic effect, while treatment with the enzymes alone also reduced the tissue damage caused by P. aeruginosa infection.

### Histopathological analyses.

**FIG 3:**
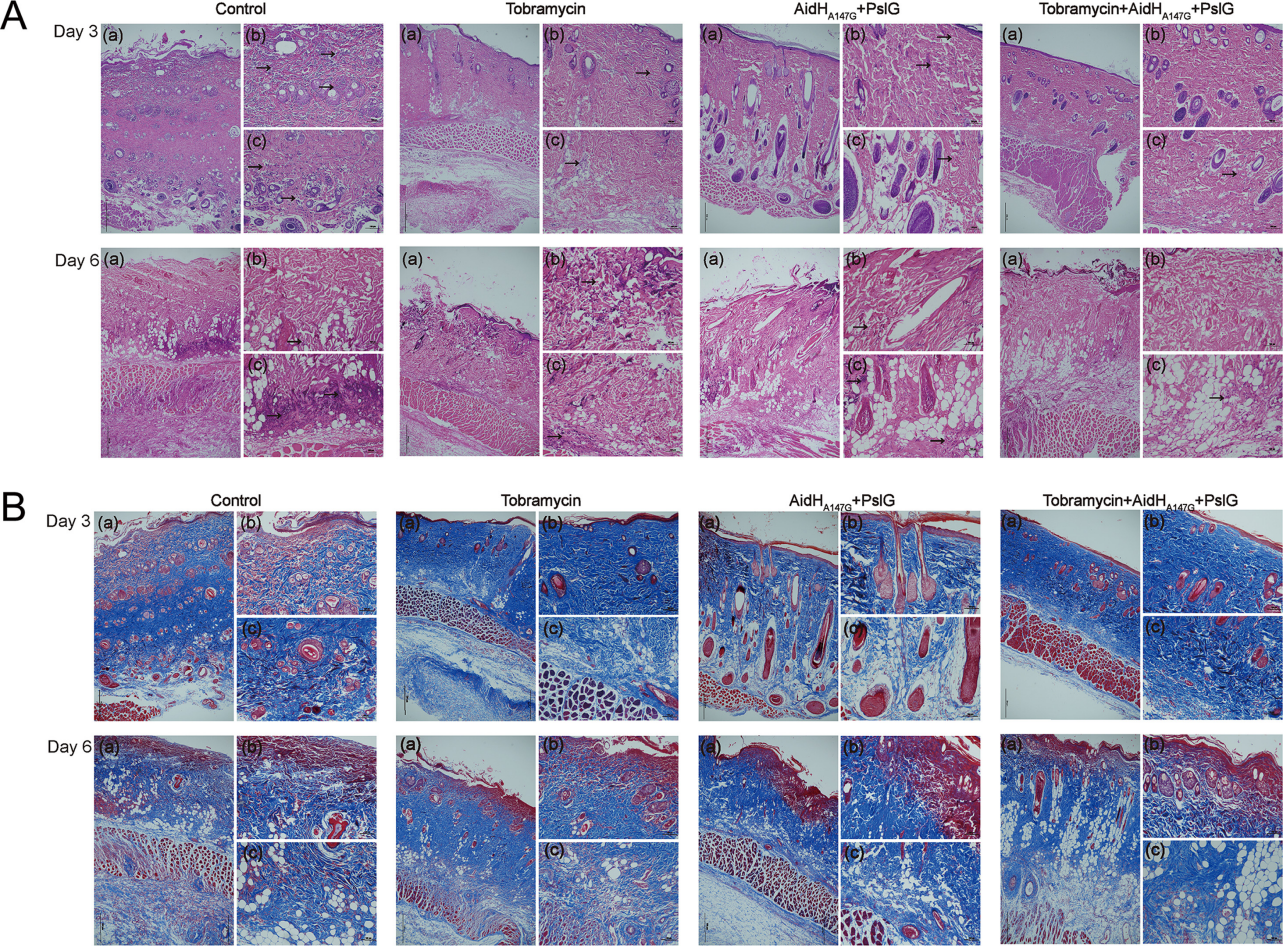
Histological analysis of skin wounds. (A) H&E stained sections of skin tissues from treated burn wounds at days 3 and 6. Black arrows point to inflammatory cells. (B) Masson stained sections of skin tissues from treated burn wounds at days 3 and 6. (a) Main image at 4 ×. (b) Higher magnification view of epidermis image at 10 ×. (c) higher magnification view of dermis and subcutaneous tissues image at 10 ×. The combination treatment group showed the least inflammatory cell infiltration.

On the third day after scalding, hematoxylin and eosin (H&E) staining (Fig. 3A) showed a large infiltration of inflammatory cells around the superficial dermis, collagen fibers, and glands in the control group. In the enzyme treatment and the tobramycin groups, inflammatory cells were observed in the subepidermal and fascial layers of the injury; however, only a few were present in the deep collagen fibers of the combination group. On the sixth day after scalding, the inflammation extended to the deep dermis and subcutis. Among the rats in the control group, a large number of inflammatory cells were found in the deep dermis and subcutaneous tissues. Compared to the control group, there was no variation in location of inflammatory cells in the enzyme treatment and tobramycin groups, but the level was reduced. The combination treatment had the least inflammatory cell infiltration, suggesting it had the optimal effect in terms of alleviating the local inflammatory response caused by the bacteria.

Masson staining (Fig. 3B) was performed to compare the extent of wound recovery in control and treatment groups. Three days after scalding, the epidermal tissues in all four groups appeared damaged, and the collagen fibers were neatly aligned. Compared to the three treatment groups, the skin damage was more severe in the control group owing to a large infiltration of inflammatory cells into the superficial dermis. On the sixth day, red-stained tissue appeared in the superficial dermis of all four groups. The red-stained tissue in the control group was similar to the blue-stained collagen fibers, again being neatly aligned. The red-stained tissue in the superficial dermis of the other three groups was uniform and failed to exhibit the arrangement of collagen fibers, which would correspond to burn scabs during the burn healing process.

### Analysis of wound biofilm formation.

**FIG 4:**
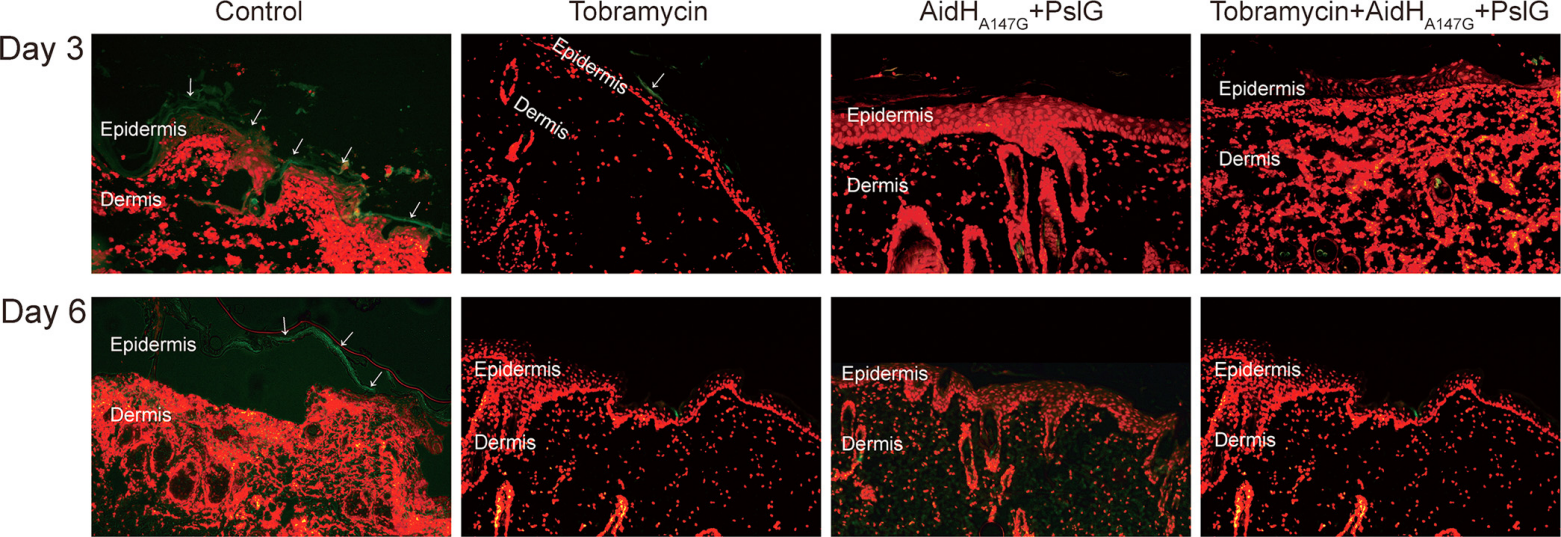
Fluorescence staining for the relationship between traumatic biofilm and tissue at days 3 and 6. The extracellular polysaccharide components of biofilms were stained with FITC-ConA in green, and the tissue nuclei were stained with PI in red. The white arrow points to biofilm. After enzyme intervention, biofilms were difficult to observe on the wound surface.

Fluorescence staining (Fig. 4) was performed to examine the relationship between traumatic biofilm and tissue. On the third day after scalding, the formation of P. aeruginosa biofilm was evident on the necrotic epidermis in the control group, but less biofilm was evident in the tobramycin group. The addition of enzymes reduced the formation of biofilm further. Little biofilm was observed in both the enzyme treatment and combination treatment groups. On the sixth day after scalding, biofilm was decreased in the control group. In other three experiment groups, biofilm formation was also difficult to observe. The results indicate that the enzyme intervention prevented P. aeruginosa biofilms formation in scald wounds.

### Electron microscope analysis.

**FIG 5:**
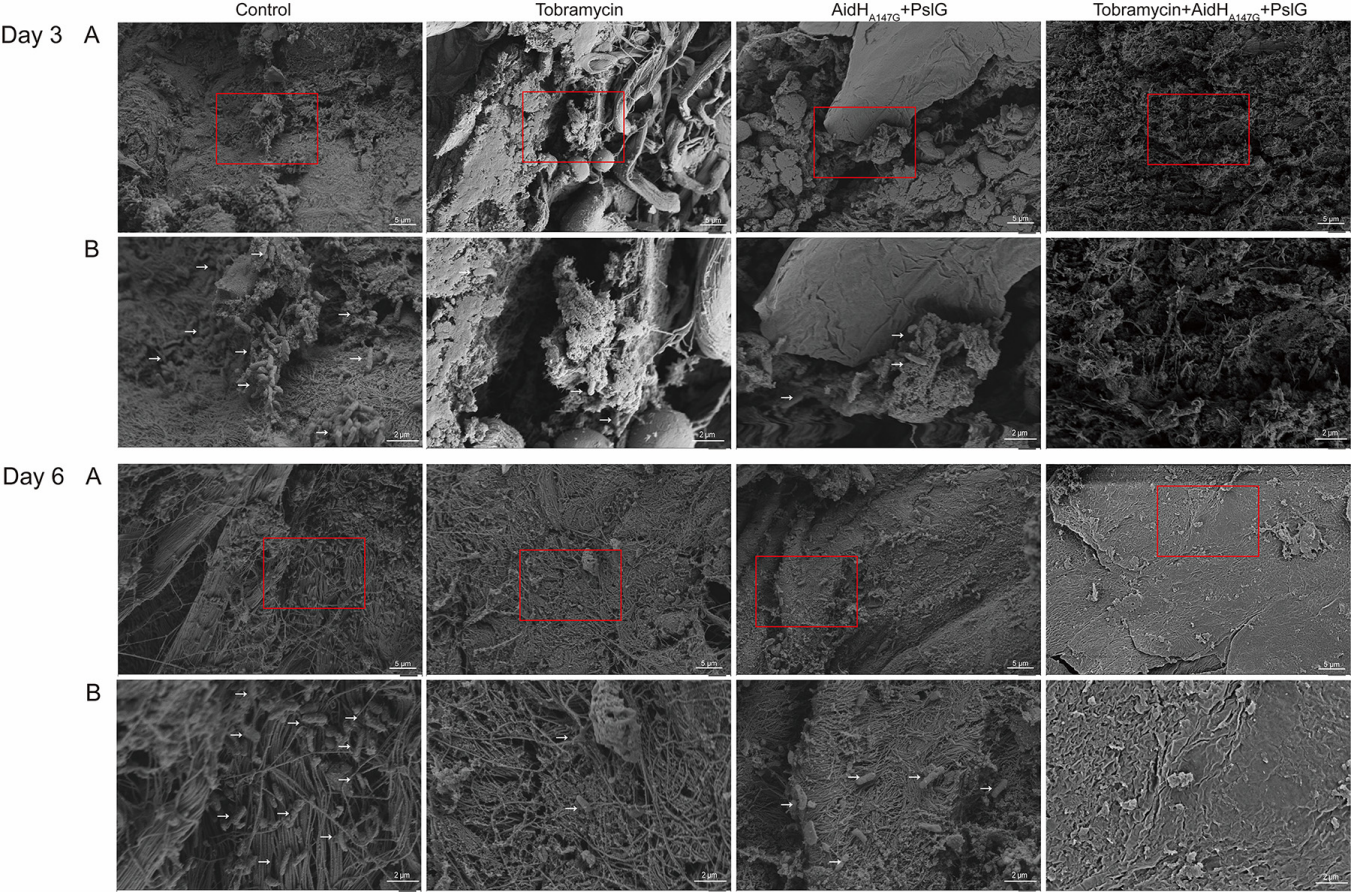
Scanning electron microscope images of the scalded wound surface at days 3 and 6. (A) Wound surface image at 2000 ×. (B) Images showing the magnified regions were indicated by red boxes in (A). The images is magnified at 6,000 ×. P. aeruginosa in the form of rods marked by white arrows. The control group presented numerous rod bacterium and microcolonies. In the combined treatment group, the presence of rod bacterium was poorly located on the wound surface.

Scanning electron microscope was conducted to observe the attachment of P. aeruginosa and biofilm to the surface of the scald wounds directly (Fig. 5). On the third day after scalding, a large number of rod bacterium was observed on the surface in the control group, either in a diffuse distribution or forming microcolonies, indicating the formation of biofilm. Compared with the tobramycin group, those in the enzyme group had a higher bacterial bioburden on the wound surface, but no microcolonies were observed. This result indicates that the enzymes intervention had an anti-biofilm effect. For the combined treatment group, the presence of rod bacterium was difficult to observe on the wound surface. On the sixth day after scalding, numerous rod bacteria were still adhering to the wound surface in the control group. Compared with the third day, rod bacteria were still present on the surface in the tobramycin and enzyme groups, whereas the surface was flat. Compared with the situation in the other three groups, the combined treatment group showed the lowest bacterial load and the optimal wound recovery.

### Expression of the QS system genes of P. aeruginosa on the wound surface.

**FIG 6:**
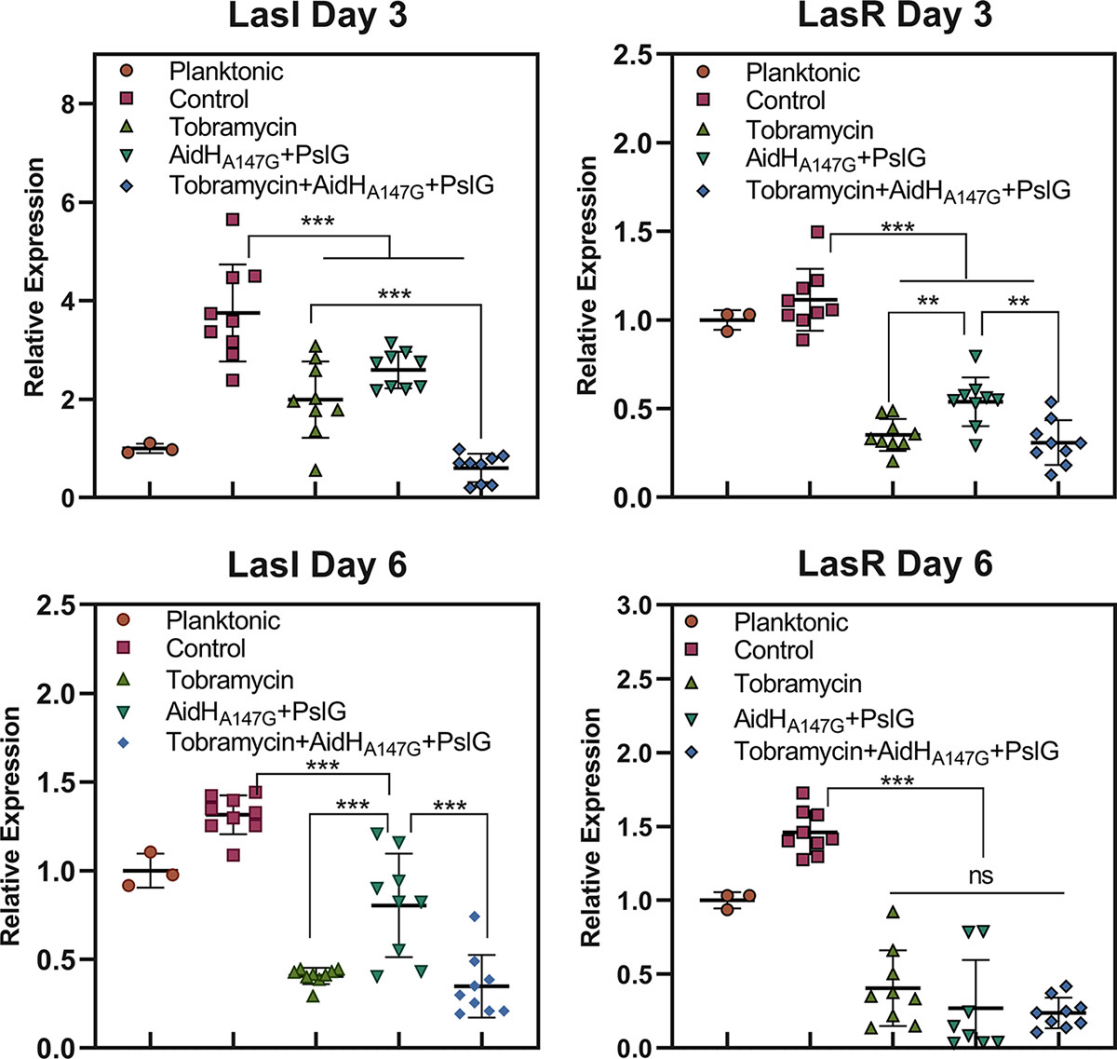
Las system genes expression of P. aeruginosa on the burn wounds at days 3 and 6. The results of the different intervention groups shown as a fold change of the planktonic inoculum. NS, no significance; *, *P* < 0.05; **, *P* < 0. 01; ***, *P* < 0.001.

*In vitro*, AidH_A147G_ could inhibit virulence factors and biofilm formation through the quorum quenching effect (20). To detect the effect *in vivo*, total RNA from wound tissues was extracted and subjected to real-time PCR. The abundance of the Rhl system gene in the tobramycin and combination treatment groups was too low to be detected. Therefore, we demonstrated the expression patterns of quorum quenching using the Las system, with the results shown as a fold change of the planktonic inoculum (Fig. 6). On the third day after scalding, significant variations in LasI expression were observed across the four groups. Compared with planktonic inoculum, the control group showed a 3.8-fold increase in LasI expression, while the enzyme treatment group showed a 2.6-fold increase over the planktonic inoculum. LasI expression in the tobramycin group showed a 1.8-fold increase over the planktonic inoculum, higher than the combination treatment group, which showed a 0.5-fold decrease in expression compared with the planktonic inoculum. The expression of LasR in the three experimental groups was lower than that in the control group. However, the combination treatment group and tobramycin group showed no differences in expression. This result proved that the enzymes played a role in the quorum-quenching. On the sixth day, the expression of Las system genes in the enzyme-treatment group was lower than that in the control group, showing a 0.8-fold decrease and a 1.3-fold increase in expression compared with the planktonic medium, respectively. No significant differences were observed between the combination treatment group and tobramycin group that may have been associated with wound healing and infection reduction.

### Bacterial load quantification *in vivo*.

**FIG 7:**
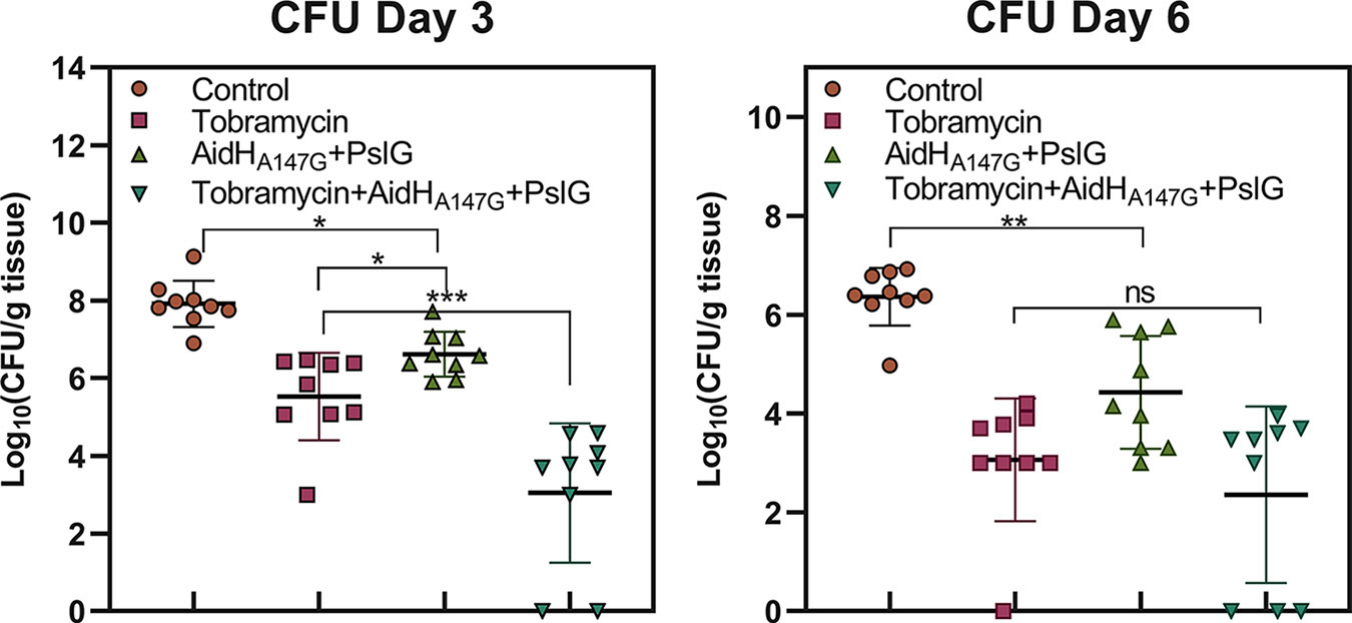
Bacterial load in the burned tissue of different intervention groups at days 3 and 6. Tissue bacterial load was plotted as log_10_ (CFU/g wound tissue). NS, no significance; *, *P* < 0.05; **, *P* < 0. 01; ***, *P* < 0.001.

Because the inhibition of biofilm enhances the effect of the immune system as well as the antibacterial effect of antibiotics, it has been assumed that the combined enzymes exert antibacterial and adjuvant antibiotic effects *in vivo* (4, 5). On the third day after scalding, the counts of P. aeruginosa for the control group were approximately 10^8^ CFU/g tissue, significantly higher than the enzyme treatment group, which had counts of 10^7^ CFU/g tissue (Fig. 7). In addition, because the combined enzymes also play a significant role as auxiliary therapy in conjunction with antibiotics *in vivo*, the CFU counts of the combination treatment group (10^3^ CFU/g tissue) were significantly less than the tobramycin group (10^6^ CFU/g tissue). On the sixth day, the bacterial load was less than on the third day in all groups, indicating that the infections were in remission. The enzyme treatment group (10^6^ CFU/g tissue) was lower than the control group (10^4^ CFU/g tissue). However, there was no discrepancy between the combination treatment group and the tobramycin group. This finding suggests that, whether used alone or in conjunction with antibiotics, the combined enzymes can reduce the tissue bacterial load and, thus, infection.

### Analysis of the tissue inflammation reaction.

**FIG 8:**
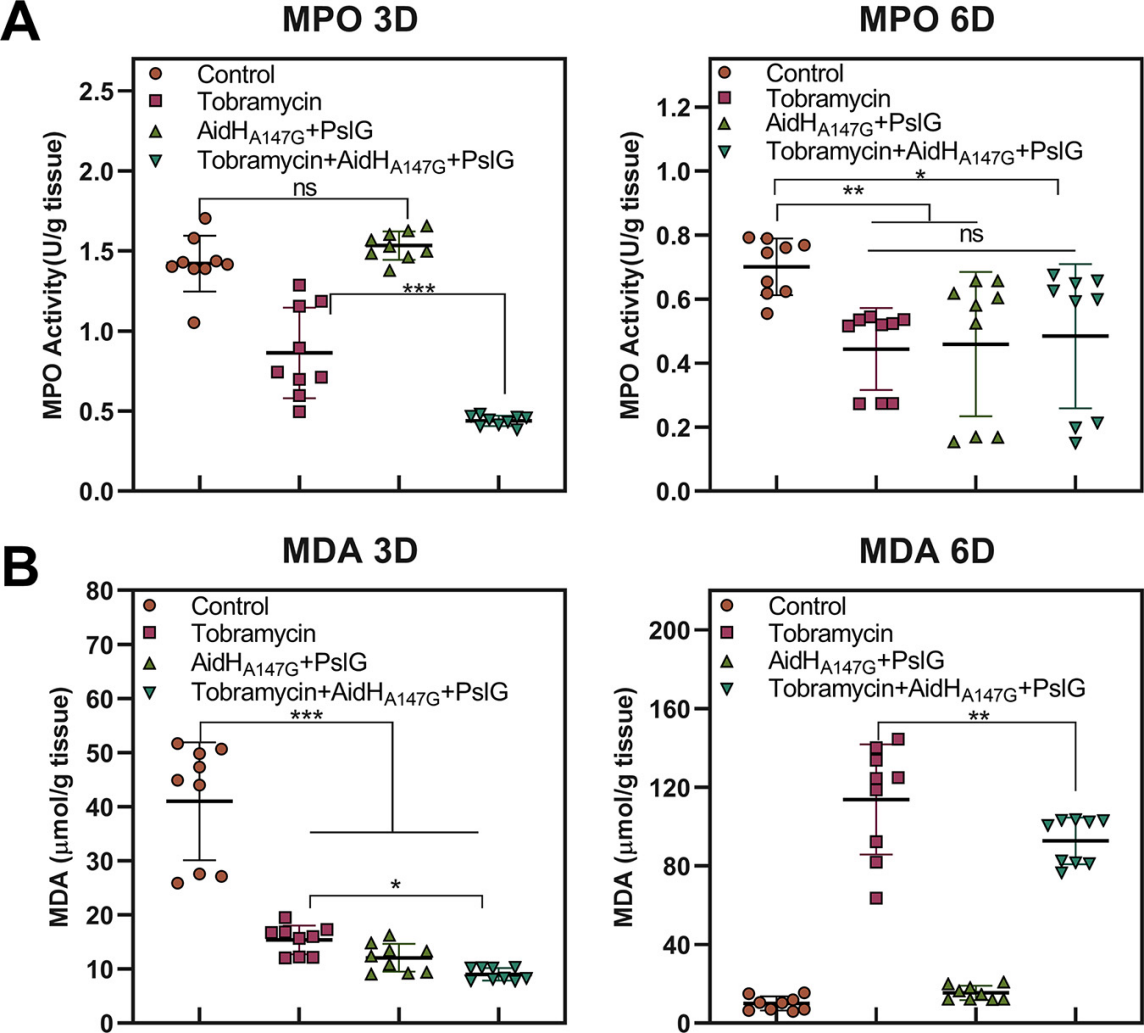
Analysis of the inflammatory reaction in burned tissue at days 3 and 6. (A) Represents the level of MPO in tissues of different intervention groups. (B) Represents the level of MDA in tissues of different intervention groups. NS, no significance; *, *P* < 0.05; **, *P* < 0. 01; ***, *P* < 0.001.

To evaluate the inflammatory reaction, MPO and MDA analysis were performed on the third and sixth days. As Fig. 8A, on the third day, there was no difference in the MPO activity between the control and enzyme treatment groups, in both of which it was approximately 1.5 U/g tissue. The addition of tobramycin treatment led to a decrease in the MPO activity, as well as the activity of the combined treatment group (0.44 U/g tissue) was lower than the tobramycin group (0.91 U/g tissue). On the sixth day, consistent with the results of the bacterial load quantification, the MPO activity was reduced in all groups relative to the third day. However, except the control group (0.64 U/g tissue), no significant differences were observed among the three experiment groups (about 0.5 U/g tissue). Regarding the MDA activity (Fig. 8B), on the third day after scalding, the control group (41 μmol/g tissue) was higher than the three experimental groups, and the combined treatment group (9 μmol/g tissue) was lower than the tobramycin group (15 μmol/g tissue). However, obvious increase in tissue MDA content was seen on sixth day with tobramycin and combination therapy. These results may be related to the scabbing of wounds (21). Therefore, we conclude that the use of the combined enzymes reduced the inflammatory damage at the wound site.

### Analysis of the systemic inflammatory response.

**FIG 9:**
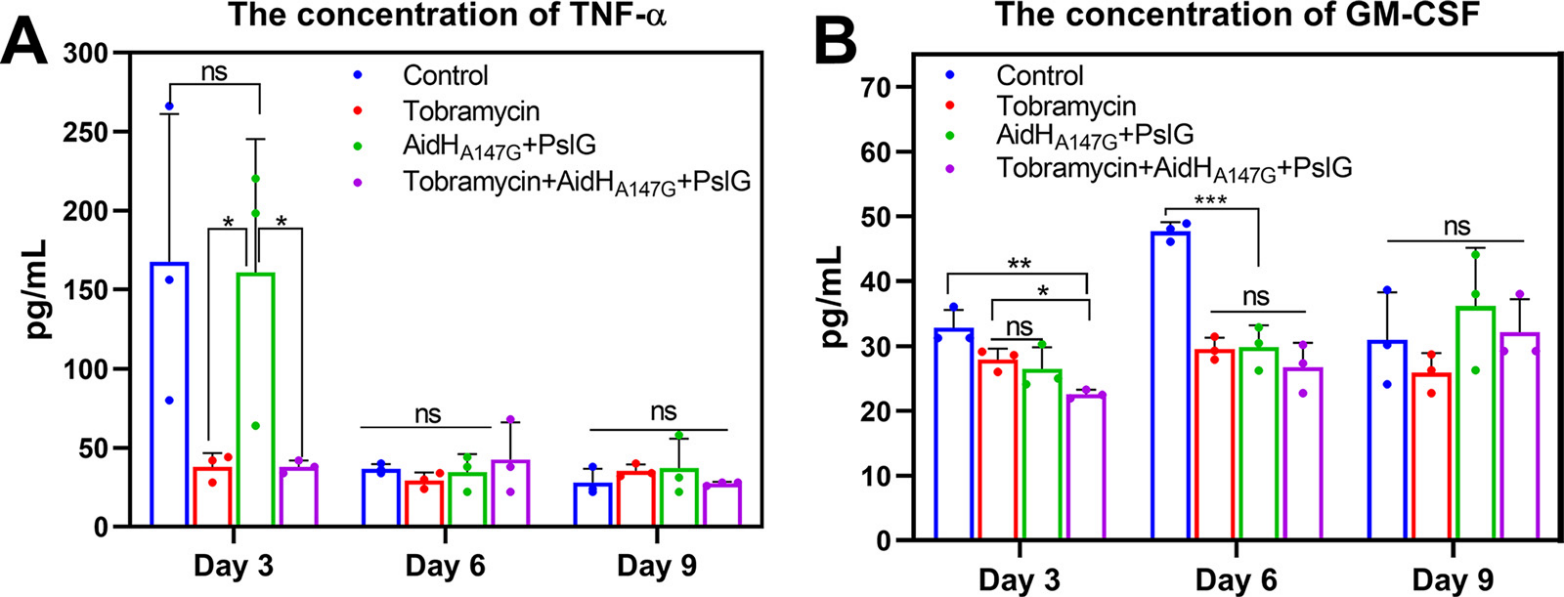
Levels of cytokines in rats plasma for different intervention groups at days 3, 6, and 9. (A) Represents the concentration of TNF-α of different intervention groups. (B) Represents the concentration of GM-CSF of different intervention groups. NS, no significance; *, *P* < 0.05; **, *P* < 0.01; ***, *P* < 0.001.

Burns and infections elicit a systemic inflammatory response that includes the release of various cytokines. As Fig. 9A shows, the concentration of TNF-α in the plasma of the rats increased significantly in the control and enzyme treatment groups (about 160 pg/mL) compared with the tobramycin and combination group, on the third day after scalding. On the sixth and ninth day, the concentrations of TNF-α remained at low levels in all four groups. For the granulocyte-macrophage colony-stimulating factor (GM-CSF) (Fig. 9B), on the third day after scalding, the rats in all four groups released low levels of GM-CSF. Nevertheless, the combined treatment group (about 22 pg/mL) was slightly lower than the other groups (about 30 pg/mL). Compared with the control group (48 pg/mL), the concentrations of GM-CSF remained at lower levels in the three treatment groups (about 0.5 U/g tissue) on the sixth and ninth days. Collectively, these results suggested that the enzymes treatment attenuated the systemic inflammatory response of the host.

## DISCUSSION

The characteristic property of the biofilm lifestyle poses great challenges for the use of conventional antimicrobials, so there is a need for multitargeted or combinatorial therapies (17, 22, 23). In our previous *in vitro* study, a combined-enzyme strategy was proposed for the first time to prevent biofilm formation and simultaneously break down preformed biofilms (20). The combined enzymes strategy facilitated the anti-biofilm effect and bacterial killing by many common antibiotics. Burn wounds can create significant damage to human skin, and 75% of burn deaths are caused by infection (24). Within burn wound exudates, opportunistic pathogens can proliferate and form biofilms (8). In order to make combined-enzyme strategy more robust and practical for clinical applications, we sought to gain further insight into the efficacy of combined enzymes (AidH_A147G_+PslG) in treating burn wound infections. By assessing the severity of the infection and inflammation, it demonstrated that simultaneous provision of antibiotics and anti-biofilm enzymes yields superior clinical benefits during burn infections.

However, only few enzyme-based therapeutic options are available to treat infections on scald model. Parul et al. demonstrated that lactonase prevented systemic spread of P. aeruginosa through burned skin and reduced mortality (25). Compared with the use of one strategy, the combination of lactonase and ciprofloxacin treatment resulted in minimal toxicity and inflammatory response to P. aeruginosa. In addition, Masarra et al. formulated recombinant N-acylhomoserine lactonase (Ahl-1) as a hydrogel (26). Consistent with the report of Parul et al., Ahl-1 hydrogel reduced the systemic spread of the multidrug-resistant P. aeruginosa infection and decreased the mortality rate. However, none of these studies of lactonase discussed the effect of the intervention on biofilm associated with the burn wound. Rather, the focus was on the *in vitro* effects of enzymes that directly disperse biofilms at the wound site, with insufficient *in vivo* research having been conducted thus far (27, 28). Consistent with the *in vitro* results, we demonstrated in the present study the biofilm inhibition and quorum-quenching effects of the combined enzymes *in vivo*. This is the first study to demonstrate the quorum-quenching effects at the level of gene expression in burn wounds using the rat model. We also showed the combined-enzyme treatment to be safe.

In combination with traditional antibiotic treatments, honey (29), herbal products (30, 31), phage application (32–34), synthetic immunomodulators (35), photodynamic therapy (36), and antibacterial materials (9, 37, 38) have been used to treat scald infections caused by P. aeruginosa. All these treatment options have been reported to reduce the damage caused by the infection as well as the mortality rate. However, most of these methods are bactericidal and bacteriostatic. Such methods to control the growth of pathogens tend to lead to the development of drug resistance. Because neither quorum-quenching nor biofilm disruption affects the growth of P. aeruginosa (18, 19, 39), these methods do not lead to resistance. Except for enzymes, azithromycin has also been used to quench quorum-sensing in scalded infections with P. aeruginosa (40). In practice, however, the use of azithromycin may cause the side effects of ototoxicity and, again, drug resistance (4). Nitric oxide can induce the dispersal of P. aeruginosa biofilms through the induction of phosphodiesterases; thus, Jiawen et al. demonstrated that magnetic nanoparticles IO^@^PMB-SNO NMs grafted thiolated nitric oxide can disrupt biofilms and promote wound healing (41). However, it is necessary to remove the nanomaterials from the wound after use. In comparison with previous studies, our combined-enzyme approach has the advantages of the targeted inhibition of virulence factors and the disruption of biofilms, delivering a superior complementary effect with both antibiotics and the immune system in fighting infections.

As part of the body’s effort to eradicate biofilm-related infections, immune cells accumulate at the wound site. However, this accumulation does not facilitate healing but instead causes inflammation to persist (42). Moreover, the Las and Rhl QS systems have been shown to be active during the early stages of bacterial growth in burn wounds as well as associated with bacterial dissemination and the expression of virulence factors (8). Thus, inflammation can be reduced by targeting QS and biofilm. MPO and MDA expression are reliable indicators of inflammation in burn wounds. MPO participates in the oxidative explosion of neutrophils (43), and the high expression of MDA delays healing (44, 45). During healing, the administration of enzymes positively influenced the levels of MPO and MDA. Moreover, the intervention with the enzymes also reduced the cytokine GM-CSF, which is associated with neutrophil proliferation and maturation. The anti-biofilm approach described here reduced inflammation, thereby promoting the healing of infected wounds.

Antibiotic resistance is a growing public health concern. The combined enzymes significantly enhanced the bacteriostatic effect of the antibiotics administered during the healing of the infected burn wounds. Because the enzymes target biofilms, this approach significantly reduces the possibility of drug resistance and represents a promising method for the treatment of scald infections. The use of the enzymes alone also significantly reduced the bacterial content and tissue damage at the burn site. This result suggests that the enzymes may serve as a substitute for antibiotics to treat nonserious infections associated with limited tissue damage.

In conclusion, the use of AidH_A147G_ and PslG in combination is a potentially promising therapy for treating P. aeruginosa biofilm on burn wounds. The combined enzymes can enhance the activities of antibiotics, thereby reducing inflammation, the extent of tissue damage, and levels of bacteria. Moreover, the combined enzymes produced certain therapeutic effects even without co-treatment with antibiotics. Future studies could, accordingly, explore the application of the combined enzymes in various P. aeruginosa infection models.

## MATERIALS AND METHODS

### Ethics statement.

The experiments using animal models were performed in accordance with Anhui Medical University Institutional Animal Care and Use Committee (IACUC) ethics approval and guidelines (protocol no. LLSC20210766).

### Chemicals and materials.

The RNA stabilization mix, RNeasy MinElute Cleanup kit was purchased from Qiagen (Hilden, Germany). TRIpure Reagent was purchased from Aidlab (Beijing, China). PrimeScript RT reagent kit and TB Green Premix Ex Taq II were purchased from TaKaRa (Dalian, China) and used according to the manufacturer recommendations. The other chemicals and reagents were of analytical grade and purchased from commercial sources unless otherwise stated. Propidium iodide, and FITC-ConA were purchased from Sigma-Aldrich (St. Louis, MO, USA.). Rat TNF-α ELISA Kit and Rat GM-CSF ELISA Kit were purchased from ELK Biotechnology (Wuhan, China). MPO Detection Kit and MDA Detection Kit were purchased from Bestbio (Shanghai, China).

### Cloning, expression, and purification of N-acylserine lactase AidH_A147G_ and glycosyl hydrolase PslG.

AidH_A147G_ and PslG were cloned into an expression plasmid as described in our previous study (19). Briefly, Escherichia coli BL21(DE3) (TOLOBIO, Shanghai, China) was transformed with the expression plasmid and grown in LB medium with 50 μg/mL kanamycin. When the cells reached an OD_600_ of 0.5 to 0.6, protein expression was induced by isopropyl-β-d-thiogalactopyranoside at a final concentration of 0.1 mM at 30°C for 10 h. The cells were harvested and disrupted by sonication. Both proteins were purified using nickel-affinity chromatography. The purified proteins were dialyzed in sodium phosphate buffer (50 mM, pH 6.8) for 3 h to reduce the imidazole concentration.

### Cell toxicity.

We used the CCK8 assay and erythrocyte fragility to assess the cell cytotoxicity of AidH_A147G_ and PslG. For the CCK8 assay, human dental pulp cells used in this study were collected in our laboratory. The cells were maintained in Dulbecco’s modified Eagle medium (DMEM) supplemented with 10% fetal bovine serum (FBS) and 1% P/S that was replaced every 2 days. For the cytotoxicity assay, the cells were seeded in 96-well culture plates and incubated for 12 h and 24 h, followed by incubation with fresh DMEM media containing various concentrations of AidH_A147G_ (a high concentration of 800 μg/mL, a middle concentration of 200 μg/mL, and a low concentration of 50 μg/mL), PslG (a high concentration of 200 μg/mL, a middle concentration of 50 μg/mL, and a low concentration of 12.5 μg/mL), and AidH_A147G_ + PslG (AidH_A147G_ and PslG concentrations were consistent with previous). The various concentrations of enzymes were diluted using PBS, and equal volumes of PBS were added to the control group. At the various time points, 10 μL CCK-8 solution was added to the ongoing cultures. After 2 h of incubation, the quantities of viable cells were tested spectrophotometrically at 450 nm using a multiscanner.

To test for erythrocyte fragility, we obtained blood from rats by cardiac puncture. The erythrocytes were isolated by centrifugation at 1,000 g for 10 min at 4°C and then washed three times and resuspended at a concentration of 2% in PBS. Next, 150 μL Triton X-100 (positive control), 150 μL PBS (negative control), 200 μg/mL AidH_A147G_, 50 μg/mL PslG, and 200 μg/mL AidH_A147G_ + 50 μg/mL PslG were added to the erythrocyte solution. After 30 min of incubation, the suspension was centrifuged at 1,000 g for 10 min at 4°C. The supernatant was aspirated and detected spectrophotometrically at 540 nm using a 96-well plate and a multiplex scanner. The percentage (%) of hemolysis was established as

$$\text{Hemolysis}(\text{\%})=\frac{\left({\text{Absorbance}}_{\text{sample}}-{\text{ Absorbance}}_{\text{PBS}}\right)}{\left({\text{Absorbance}}_{\text{Triton X}-100}-{\text{ Absorbance}}_{\text{PBS}}\right)}\times 100\%$$

### Rats burn and infection model.

To establish the rat burn and infection model, 10-week-old male Sprague-Dawley rats weighing 320 to 400 g were used and anesthetized with 30 mg/kg sodium pentobarbital. The dorsum was shaved and depilated with 10% sodium sulfide. For the induction of second-degree burns, a YLS-5Q hot device (90°C) consisting of a circular steel rod 2 cm in diameter was applied to the dorsal area for 15 s to create three scald wounds on the back. H&E staining histology demonstrated such burn experimental results (Fig. S1). After overnight culture, P. aeruginosa PAO1 was centrifuged and resuspended in 0.9% NaCl. Immediately after the scalding, each wound was inoculated with 1*10^7^ CFU of P. aeruginosa. Four groups of six rats were treated with 0.9% NaCl (as a control), 30 μg/wound tobramycin, 100 μg/wound AidH_A147G_ + 25 μg/wound PslG, and 30 μg/wound tobramycin + 100 μg/wound AidH_A147G_ + 25 μg/wound PslG, respectively. The wounds were covered with surgical dressings and sterile nonwoven gauze. The treatments were injected between the surgical dressing and the wound surface. The treatments were applied every 24 h. The rats were sacrificed at three experimental endpoints (3, 6, and 21 days), and their tissues were extracted.

### Histological analysis.

The scalded tissue was fixed with 4% paraformaldehyde, embedded in paraffin, and then sectioned. Paraffin-embedded sections were stained with H&E or Masson’s trichrome reagents after dewaxing using xylene and ethanol. The stainings were performed to examine the degree of inflammation and injury. To examine the relationship between biofilm and tissue, propidium iodide and FITC-ConA were used for fluorescence staining. For cell staining, the sections were stained with propidium iodide for 15 min and then washed thrice with PBS. For the biofilm staining, the sections were stained with FITC-ConA for 30 min and then washed thrice with PBS. The anti-fluorescence quencher served to seal the sections, and we then collected the images under a fluorescence microscope.

### Scanning electron microscopy analysis.

To observe P. aeruginosa infection in the burn wounds, we minced biopsy tissue into 4-mm cubes and fixed the samples with 2.5% phosphate-buffered glutaraldehyde at 4°C for 24 h. After fixation, the samples were washed in pH 7.2 sodium phosphate buffer (100 mM) and then dehydrated in a graded series of cold ethanol/water mixtures (30%, 50%, 70%, 80%, 90%, and 100% ethanol) for 15 min each. After dehydration, the samples were critical-point dried and sprayed with gold particles. We then scanned and imaged the samples in a scanning electron microscope (ZEISS GeminiSEM 300).

### Bacterial load quantification *in vivo*.

The cut skin included the epidermis, dermis, and subcutaneous tissues. For bacterial load quantification, 0.1 g of tissue from each of the wounds was used. Tissue homogenized with 0.5 mL cold normal saline with a high-speed tissue grinder. The samples were serial diluted with normal saline and plated on P. aeruginosa isolation agar. After overnight incubation, the number of monoclonal strains on the plates was counted and plotted as log_10_ (CFU/g tissue) ± the standard deviation.

### Quantitative RT-PCR for expression of P. aeruginosa QS system genes expression.

The cut skin included the epidermis, dermis, and subcutaneous tissues. For QS system gene expression quantification, 0.2 g of tissue from each of the wounds was taken. Tissue homogenized with 1 mL RNA stabilization mix using a high-speed tissue grinder. The TRIzol method served to extract the total RNA from the tissues and bacteria and the RNeasy MinElute Cleanup Kit to purify it. As recommended, we performed the real-time PCR using a PrimeScript RT reagent kit and TB Green Premix Ex Taq II. The qPCR primers used in this study are consistent with those used in our previous study (19).

### MPO and MAD assay.

Samples were detected in accordance with the myeloperoxidase assay kit and malondialdehyde assay kit collecting 0.05 g of tissue from each of the wounds for the assays. As before, the cut skin included the epidermis, dermis, and subcutaneous tissues. MPO activity of the skin tissue was detected by measuring the 3,3′-dimethoxybenzidine oxidation by H_2_O_2_ at 460 nm. MPO activity was expressed as units per gram of wet tissue, with one unit indicating that the enzyme degraded 1 μmol of H_2_O_2_ at 37°C. The MDA content of the skin tissue samples was detected by measuring the chromogen obtained from the reaction of MDA with 2-thiobarbituric acid and expressed as μM/g of wet tissues. The detection process was performed following the instruction leaflet.

### Detection of serum inflammatory cytokines.

To analyze the levels of inflammatory cytokines, blood was collected by cardiac puncture on days 2 and 7, and by tail clipping on day 9 in anticoagulated tubes containing EDTA-Na_2_. The samples were centrifuged at 4°C for 10 min at 1,000 g to prepare the plasma. ELISA served to measure the levels of TNF-α and GM-CSF in the plasma samples from the various treatments following the manufacturer’s protocol.

### Statistical analysis.

IBM SPSS Statistics 25 was relied to analyze the data. Results were compared using one-way analysis of variance (ANOVA) with Tukey’s multiple-comparison test to identify significant differences between the experimental groups. Significance was accepted when the *P* value was ≤0.05. GraphPad Prism 7.03 served to plot the data. All experiments were done in triplicates. In the figure, *indicates *P* < 0.05, ** indicates *P* < 0. 01, and *** indicates *P* < 0.001.
