# Supplementary material for: Anti-Biofilm Enzymes-Assisted Antibiotic Therapy against Burn Wound Infection by Pseudomonas aeruginosa
Source: Antimicrob Agents Chemother. 2023 Jun 5;67(7):e00307-23. doi: 10.1128/aac.00307-23 (PMC10353415; doi:10.1128/aac.00307-23)
Supplement: Supplemental file 1 — Fig. S1. Download aac.00307-23-s0001.docx, DOCX file, 0.8 MB [file aac.00307-23-s0001.docx]

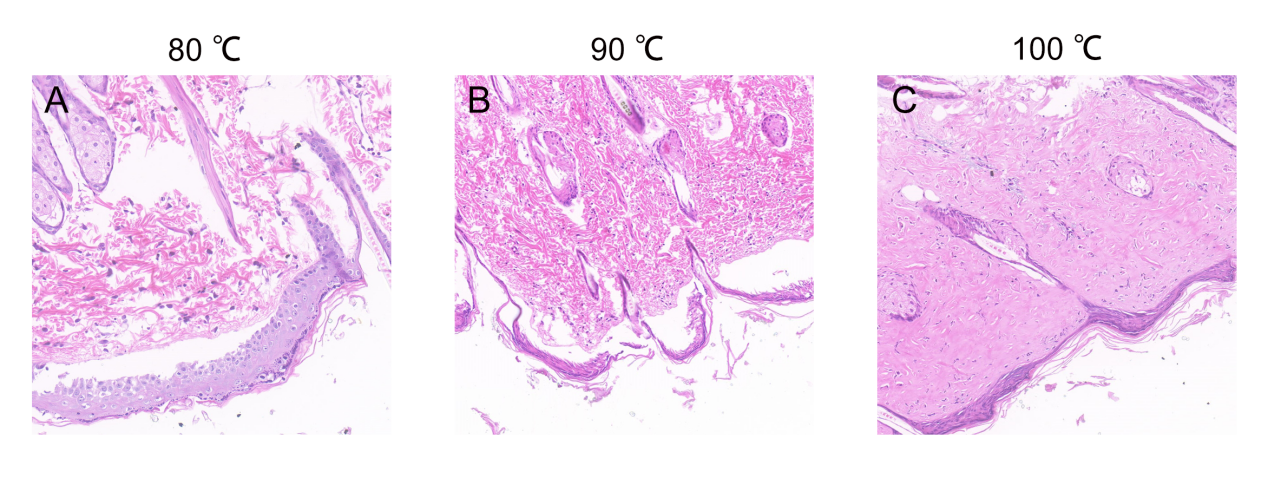


Figure S1. H&E stained sections of skin tissues from burn wounds. (A) The skin was scalded at 80 ℃ for 15 s. (B) The skin was scalded at 90 ℃ for 15 s. (C) The skin was scalded at 100 ℃ for 15 s.
